# Optimization of Hybrid Ink Formulation and IPL Sintering Process for Ink-Jet 3D Printing

**DOI:** 10.3390/nano11051295

**Published:** 2021-05-14

**Authors:** Jae-Young Lee, Cheong-Soo Choi, Kwang-Taek Hwang, Kyu-Sung Han, Jin-Ho Kim, Sahn Nahm, Bum-Seok Kim

**Affiliations:** 1 Icheon Branch, Korea Institute of Ceramic Engineering & Technology, Icheon 17303, Korea; wony0827@naver.com (J.-Y.L.); 180914@kicet.re.kr (C.-S.C.); kthwang@kicet.re.kr (K.-T.H.); kh389@kicet.re.kr (K.-S.H.); 2 Department of Material Science and Engineering, Korea University, Seoul 02841, Korea; wony0827@korea.ac.kr; 3 Thin Lamination Board (TLB), Sinwon-ro, Danwon-gu, Ansan-si 15602, Korea; bskim@tlbpcb.com

**Keywords:** ink-jet 3D printing, IPL sintering, rheological properties, photo-curable nano SiO_2_ ink, IPL-sinterable nano Cu ink

## Abstract

Ink-jet 3D printing technology facilitates the use of various materials of ink on each ink-jet head and simultaneous printing of multiple materials. It is suitable for manufacturing to process a complex multifunctional structure such as sensors and printed circuit boards. In this study, a complex structure of a SiO_2_ insulation layer and a conductive Cu layer was fabricated with photo-curable nano SiO_2_ ink and Intense Pulsed Light (IPL)-sinterable Cu nano ink using multi-material ink-jet 3D printing technology. A precise photo-cured SiO_2_ insulation layer was designed by optimizing the operating conditions and the ink rheological properties, and the resistance of the insulation layer was 2.43 × 10^13^ Ω·cm. On the photo-cured SiO_2_ insulation layer, a Cu conductive layer was printed by controlling droplet distance. The sintering of the IPL-sinterable nano Cu ink was performed using an IPL sintering process, and electrical and mechanical properties were confirmed according to the annealing temperature and applied voltage. Then, Cu conductive layer was annealed at 100 °C to remove the solvent, and IPL sintered at 700 V. The Cu conductive layer of the complex structure had an electrical property of 29 µΩ·cm and an adhesive property with SiO_2_ insulation layer of 5B.

## 1. Introduction

The printed electronics industry using ink-jet printing technology has been in the spotlight as a next-generation technology. The ink-jet printing process is a simple process, which does not require development, exposure, and etching in the existing patterning technology. The substrate and material properties can be maintained from chemical influences. Additionally, ink-jet printing technology has many advantages of the environmentally friendly process, low production costs, and a fast production speed, because it distributes ink to only selected areas with high efficiency, resulting a minimized waste. Recently, ink-jet printing technology has been actively researching 3D printing technology, which accurately stacks various ink-type materials from multiple print heads [1,2,3,4]. The printer head of ink-jet printing has hundreds to thousands of nozzles, and each nozzle has precise and individual jetting controls. Therefore, the ink-jet 3D printing technology can effortlessly print complex multifunctional structures by printing multi-material ink with different physical and chemical properties at the same time [5,6,7,8,9]. Despite these advantages, research of ink-jet 3D printing using multiple materials still need to resolve many technical hurdles.

In order to produce precise and complex structures using the ink-jet 3D printing technology, it is necessary to control the viscosity, surface tension, and density of the ink by optimizing the ink composition. The formation of the ink drop from print heads and interaction with substrates can also significantly affect the final printed products [10,11]. In addition, ink-jet 3D printing can simultaneously print multiple functional inks and is very effective in stacking complex structures composed of multiple materials. Therefore ink-jet 3D printing is suitable for manufacturing micro-sensors and printed circuit boards composed of an insulating layer, and a conductive layer, and the development of insulating and conductive inks with excellent electrical, mechanical, and thermal properties. Also, in order to effectively apply a new functional ink to ink-jet 3D printing, it is important to optimize the rheological behavior of inks and printing conditions.

Precious metals such as gold (Au), silver (Ag), and platinum (Pt) have been mainly used as conductive ink materials, but their high price has led to research on new alternative materials. Copper (Cu) represents the materials that can replace this, but it is not easy to form electrodes due to problems such as oxidation in the air and re-oxidization during the thermal sintering process [12,13,14]. In addition, the high sintering temperature of Cu hinders the use of flexible plastic substrates such as Polyimide (PI) and polyethylene terephthalate (PET). Therefore, a technology that secures the electrical and mechanical properties is needed while rapidly sintering conductive patterns in selective areas without deformation of plastic flexible substrates or materials. The Intense Pulsed Light (IPL) sintering method using a xenon lamp has been utilized, allowing metal nanoparticles to be reduced in milliseconds at room temperature to manufacture electrodes with excellent electrical properties [15,16]. Research on IPL was mainly conducted on the sintering behavior of various electronic materials such as metals, nano carbons, ceramics, and polymer materials. However, little research was done on the IPL sintering behavior of electronic materials on functional substrates.

In this study, we investigated the ink-jet 3D printing process using multiple materials to produce electronic circuit structures. The rheological properties and jetting behaviors of photo-curable nano SiO_2_ ink for insulation layers and IPL-sinterable nano Cu ink for conductive patterns were optimized for ink-jet printing application. The printing characteristics of photo-curable nano SiO_2_ and IPL-sinterable nano Cu inks were also optimized for the successful manufacturing of complex structures. Finally, the effects of IPL sintering on the printed objects, which were produced using two different ink materials, were investigated in detail. Based on this research, we proposed a possibility that ink-jet 3D printing can be an attractive alternative to the manufacturing process of next-generation electronic products.

## 2. Materials and Methods

### 2.1. Schematic of Experimental Method

**Figure 1:**
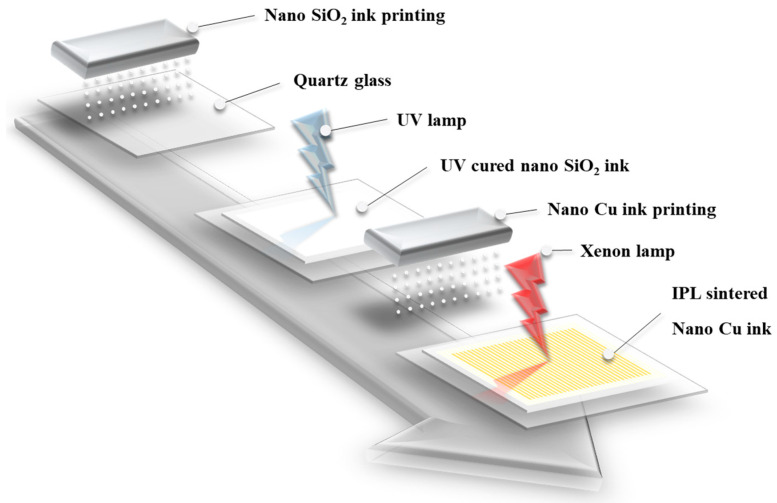
Schematic of ink-jet 3D printing and IPL sintering with photo-cured nano SiO_2_ ink and IPL-sinterable nano Cu ink.

Multi-material ink-jet 3D printing technology was used to manufacture complex structures by simultaneously printing SiO_2_ insulation and Cu conductive layers. This technology presents a simpler manufacturing method than the existing process, as shown in Figure 1.

### 2.2. Synthesis of Photo-Curable Nano SiO_2_ Ink and IPL-Sinterable Nano Cu Ink

To modify the surface properties of the SiO_2_ nanoparticles, a silane coupling agent (3-Mercaptopropyl trimethoxysilane, MPTMS, Sigma Aldrich, Munich, Germany) was added, followed by hydrolysis and condensation reactions at 50 °C for 24 h. The surface-treated SiO_2_ particles were combined with hexanediol diacrylate (HDDA, Miwon chemical, Ulsan, Korea), a photo-curable acrylic monomer, by stirring the mixture at room temperature for 3 h [17,18]. To improve the stacking printability of photo-curable nano SiO_2_ ink, distilled water was added to manufacture the modified nano SiO_2_ ink and control the contact angle of the substrates surface-modified with hydrophobicity. In the case of water-based modified nano SiO_2_ ink, distilled water and monomers were added to the existing nano SiO_2_ ink, and alkyl-diphenyl oxide disulfonate (PROCHEM) as an anionic surfactant, was added to mix two materials with different polarities effectively, and a sonication (UW-3100, Bandelin Electronics) was dispersed for 10 min. For photopolymer reactions, phylbs (2,4,6-trimethyl benzoyl) phosphine oxide (Sigma Aldrich, Munich, Germany), which reacts in the UV wavelength range of 350–430 nm, was added as a photoinitiator and dispersed for 10 min by sonication. The synthesis of conductive and IPL-sinterable nano Cu ink used commercial Cu ink (DYCOTEC, DM-CUI-5002) with a particle size of 100 nm. Polyvinylpyrrolidone (PVP, Mw = 40,000g/mol, Sigma Aldrich, Munich, Germany) was added as a surface stabilizer and a reducing agent by sonication for 30 min. Then, ethanol (EtOH, 95%, Dae-Jung, Korea) and DEG (Diethylene glycol, 99%, Dae-Jung, Korea) were added to secure the printable and stirred at room temperature for 12 h [19,20,21].

### 2.3. Multi-Material Ink-Jet 3D Printing

In order to improve the stacking printability of the photo-curable nano SiO_2_ ink, a water-repellent coating solution was coated on a glass substrate to control the contact angle. The coating solution was applied by adding perfluorooctyl-trichlorosilane (PFTS, 97%, Sigma Aldrich) to ethanol by adding 1 wt%. The quartz glass substrate was dipped in acetone and washed in the sonication for 10 min before being dried in a 75 °C oven. The dried substrate was dried for 24 h in a 75 °C oven after being dipped for 10 s in the PFTS coating solution. The contact angle of photo-curable nanoSiO_2_ ink on the hydrophobic-coated substrate was measured using an act angle analyzer (PHX300, Surface electro-optics, Korea).

Photo DSC (DSC 204 F1 phoenix, Netzsch, Germany) was used to evaluate the photopolymerization properties of the photo-curable nano SiO_2_ ink. As a light source, a UV lamp with a wavelength of 385 nm was used. The amount of light was set at 15 W/cm^2^, and the exposure time was set at 1 s. Drop watcher (Cera DW, STI) was used to analyze the ink-jet printing jetting behavior of each nano ink, and the operation condition was set to 37 to 84 V at room temperature, the rising and falling time was set to 1.5 to 2 μs, the holding time was set to 3 to 13.2 μs, the delay time was set to 1 μs, and the jetting behavior of ink was observed for 0–300 μs. In the case of the Cu nano ink, a microcircuit was implemented by controlling the drop-to-drop (D2D) distance of 50 to 200 μm. The shape of the droplet and 3D-printed structure with a photo-curable nano SiO_2_ ink and IPL-sinterable nano Cu ink was analyzed using an optical microscope (Axioscope, Zeiss, Oberkochen, Germany) and a three-dimensional laser microscope (OLS4500, Olympus, Tokyo, Japan). A three-dimensional insulation structure using modified nano SiO_2_ ink was fabricated by an ink-jet 3D printer (JW-300, Jungwon Wise, Korea) and finally designed with 10 mm horizontal and vertical widths through repeated stacking steps. The electrical resistance of the printed insulation layer was analyzed with a megger tester (SM-7110, HIOKI).

### 2.4. The Intense Pulsed Light Sintering Process

The nano Cu ink printed on a photo-cured SiO_2_ insulation layer was annealed for three hours at 100 to 300 °C and then sintered in the 400 to 800 voltage range using Intense pulsed light (IPL, myPET, semisysco, Gyeonggi-do, Korea) under room-temperature atmospheric pressure conditions. The Xenon lamps attached to the IPL sintering equipment had an energy efficiency of 99.84% in a 200–1000 nm wavelength band and 10 mm × 10 mm, and the density of energy (J/cm^2^) at a distance of 5mm on the z-axis was measured at the energy meter (Ophir Photonics, Nova II). The electrical properties of conductors were measured using 4 probe points (CMT-SR series, Changmin Tech, Gyeonggi-do, Korea). The analysis of the phase change and microstructure of annealing and photo-sealed conductors was conducted using an X-ray diffractometer (XRD, Rigaku, MAX2500VL), optical microscope (Axioscope, Zeiss, Oberkochen, Germany), and Field Emission Scanning electron microscope (FE-SEM, JEOL, JSM-690), respectively.

For the mechanical properties of the IPL-sintered conductive layer, an adhesion test was conducted based on ASTM D3359. As the film thickness was less than 50 μm, 11 × 11 cut surfaces were made at 1 mm intervals, and after attaching 3M tape, the cut surface was evenly ripped for 1 s at an angle of 180° within 90 s. If the removed area was 100 to 65%, it shall be 0 B, while 65 to 35% was 1 B, 35 to 15% was 2 B, 5 to 15% was 3 B, and if it was less than 5%, it shall be 5 B if it was not peeled by 0%.

## 3. Results and Discussion

### 3.1. Rheological Properties of Photo-Curable Nano SiO_2_ Ink

**Figure 2:**
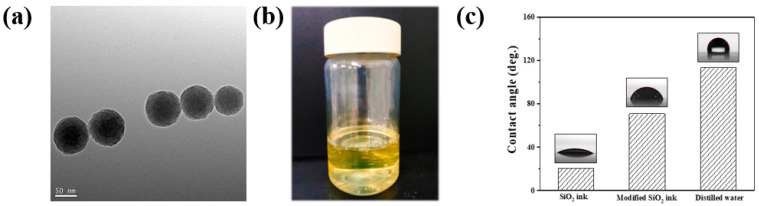
(**a**) TEM image of sol-gel synthesized nano SiO_2_; (**b**) photographs of photo-curable nano SiO_2_ ink; and (**c**) the contact angle of photo-curable nano SiO_2_ ink.

A Transmission electron microscopy (TEM) image of nano SiO_2_ particles used to synthesize photo-curable SiO_2_ ink is shown in Figure 2a,b. It has been confirmed that the SiO_2_ particles represent an elevation of about 50 to 90 nm and have a spherical shape. In order to apply SiO_2_ ink to the ink-jet printing process, it has been reported that an average particle size of about 300 nm or less must be shown to prevent blockage in the print head nozzle; most of the SiO_2_ particles used in this study showed uniform inlet and spherical shapes of less than 90 nm, which was judged to meet these requirements [20,21,22]. Figure 2c shows the contact angle of the photo-curable nano SiO_2_ ink on a hydrophobic-coated glass substrate using PFTS. If the contact angle of ink droplets after printing was low, ink spreads could occur, leading to printing resolution problems. Therefore, it was necessary to reduce the substrate surface energy to increase the contact angle between the ink droplet and the substrate [23,24]. In order to reduce the surface energy of the substrate to which ink was printed, the change in the contact angle of the SiO_2_ ink droplet was measured after the hydrophobic coating with PFTS. When the substrate was not surface treated, it was impossible to measure the contact angle due to the ink spread. After the surface treatment of the substrates, the nano SiO_2_ ink showed an initial contact angle of 20.6°, but after 5 s, it was observed that the spread was too severe to be measured. To control the droplet spreading of these nano SiO_2_ inks, water-based solvents and photo-curable monomers were combined using anionic surfactant (alkyl-diphenyl oxide disulfonate). Adding water-based solvents to the nano SiO_2_ ink was intended to maximize the hydrophobic effect on the substrates treated with PFTS. It was demonstrated that the modified nano SiO_2_ ink controlled the spread of ink significantly and increased the contact angle to 70.9°.

The rheological properties were optimized to secure the ink-jet printability of the synthesized modified nano SiO_2_ ink, as represented in Table 1. The most influential property in the jetting behavior of the nano SiO_2_ ink was the surface tension and viscosity. Based on this, the Ohnesorge values of the nano SiO_2_ ink and modified nano SiO_2_ ink were calculated using the calculated Ohnesorge number, and the results were found to be suitable for jetting at 5.82 and 7.50, respectively. At this time, photo-curable SiO_2_ ink was manufactured by adding HDDA and photo-initiator to 20 wt% SiO_2_ sol coated with MPTMS. In ink-jet printing, in the typical drop-on-demand (DOD) method, it was determined that the inverse of the Ohnesorge number represented a value between 1 and 10 [25,26,27].

### 3.2. Ink-Jet 3D Printability of Photo-Curable Nano SiO_2_ Ink

**Figure 3:**
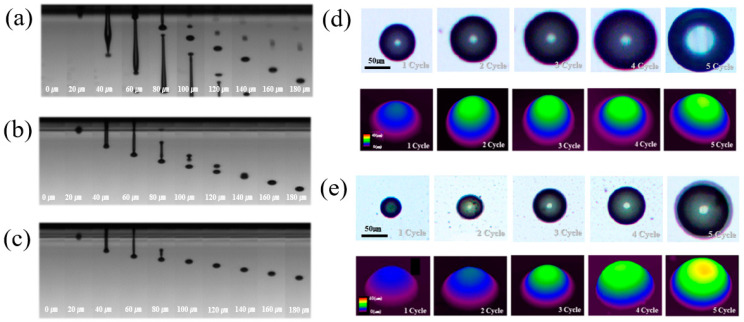
Operation conditions of ink-jet printing using (**a**) nano SiO_2_ ink before rheological optimization; (**b**) nano SiO_2_ ink; and (**c**) modified nano SiO_2_ ink after rheological optimization; microscope images of ink-jet 3D printed dot using (**d**) nano SiO_2_ ink and (**e**) modified nano SiO_2_ ink.

Figure 3 shows the result of optimizing the ink-jet operating conditions using nano SiO_2_ ink with optimized surface tension and viscosity. Figure 3a features the droplet formation behavior of nano SiO_2_ ink before optimizing the operating conditions. The operating conditions of the ink-jet head were applied voltage at 70 V, rising time and fall time at 3 μs, and maintenance time at 1 μs. Before the ink-jet operating conditions were optimized, it was possible to observe the ink being jetted insecurely from the printer head, and problems such as the long tail ink and the satellite drop occurred. This was a significant cause of the low print resolution of ink-jet printing. In Figure 3b, nano SiO_2_ ink was set to 84 V operation voltage, 2.1 μs rise and fall time, and 4.8 μs dwell time. As for the jetting behavior of the optimized modified SiO_2_ ink in Figure 3c, it was confirmed that spherical droplets were generated under the operation condition of applied voltage at 65 V, the rise and fall times at 2.4 μs, and the holding time at 7.6 μs. The smaller the volume, the more precise the printing, and the slower the liquid was jetted, the more the splash drop could be prevented. Figure 3d,e depict the printed dots of nano SiO_2_ ink and modified nano SiO_2_ ink after 5 cycles of printing in the same location. The spread of the modified nano SiO_2_ ink was suppressed, forming a more precise pattern.

### 3.3. Characterization of Ink-Jet 3D Printed Insulation Structure with Photo-Cured Nano SiO_2_

**Figure 4:**
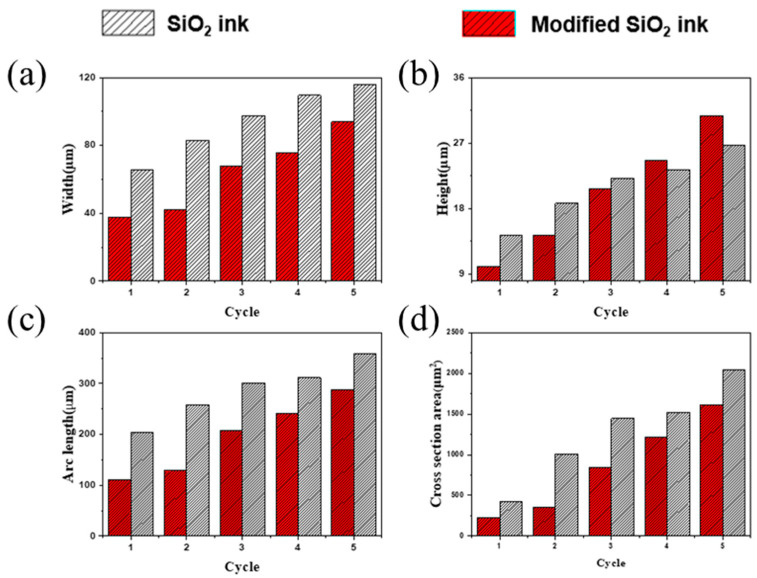
Comparative analysis results of (**a**) width, (**b**) height, (**c**) arc length, (**d**) cross-section area of ink-jet 3D printed dot using photo-curable nano SiO_2_ ink and modified nano SiO_2_ ink.

Figure 4a–d show comparative analysis results of the width, height, arc length, cross-section area of an ink-jet 3D printed dot using photo-curable nano SiO_2_ ink and modified nano SiO_2_ ink. After one cycle of printing with the nano SiO_2_ ink, the width of the ink dot was 65.86 μm, the height was 14.27 μm, the arc length was 204.42 μm, and the cross-sectional area was 423.42 μm^2^. In the case of modified nano SiO_2_, the ink dot appearance after one cycle was 37.58 μm wide, 10.06 μm high, and 111.19 μm arc length, and had a cross-sectional area of 231.93 μm^2^. It was observed that the nano SiO_2_ ink had a higher stacking efficiency than the modified nano SiO_2_ ink, which was judged to be the increase in the volume of the droplet of nano SiO_2_ ink due to increasing the internal pressure of the piezoelectric head by the high operating voltage required for the jetting of nano SiO_2_ ink. However, it was confirmed that the dot of modified nano SiO_2_ ink was higher than the nano SiO_2_ ink dot when stacked more than 4 cycles due to the superior photo-curing conversion rate of the modified nano SiO_2_ ink. Finally, the nano SiO_2_ ink dot measured after 5 cycles showed a 115.77 μm width, 26.76 μm height, 358.63 μm arc length, and 2046.1 μm^2^ cross-section area. The modified nano SiO_2_ ink dot showed a 93.95 μm width, 30.78 μm height, 288.3 μm arc length, and 1612.21 μm cross-section area. The width, arc length, and cross-sectional area of the stacked droplet showed a tendency to increase with the number of cycles. In conclusion, the modified nano SiO_2_ ink with an improved contact angle and conversion rate showed excellent stack behavior.

For the rheological properties of ink, the photopolymer reaction in curing the deposited ink before spreading is crucial. The photo-initiation plays an essential role in photopolymerization reactions by absorbing the light sources of UV wavelengths in the early stages of the photopolymerization reaction to generate free radicals and react with surrounding monomers to form a network. The photopolymerization reaction behavior according to the amount of photo-initiator added to the nano SiO_2_ ink was confirmed, and the conversion rate was calculated as follows [28,29]

(1) $$\alpha \left(t\right)\;=\;\frac{\Delta H_{sample\left(t\right)}}{\Delta H_{p}\;\times \;m}\;\times \;100$$

*α*(*t*) is the conversion rate, Δ*H_sample_*_(*t*)_ is the polymerization enthalpy for the acrylate double bond of the sample measured by photo DSC, Δ*H_p_* is the polymerization for the acrylate double bond (Δ*H_p_* = 684 kJ/kg), and *m* is the mass of the monomer.

**Figure 5:**
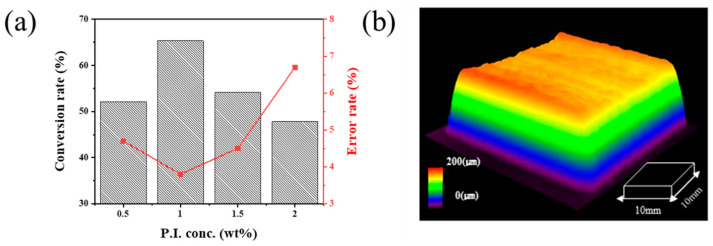
Ink-jet 3D printed insulation structure using photo-curable nano SiO_2_ ink: (**a**) Conversion rate and error rate according to photo-initiator content and (**b**) 3D image of the insulation layer using ink-jet printed nano SiO_2_ ink with 1wt% added photo-initiator.

After irradiating UV light, the reaction began after 0.1 s for the modified nano SiO_2_ ink. Initially, the photopolymer reaction rate increased rapidly, but it gradually declined above the gel point. In the case of the conversion rate, as shown in Figure 5a, the concentration of the photo-initiation agent was adjusted from 0.5 to 2.0 wt%, and the optimized concentration was 1 wt%. The modified nano SiO_2_ ink showed a photopolymerization conversion rate of 65.3% and a printing error rate of 3.8%. As shown in Figure 5b, a three-dimensional insulation layer using photo-curable modified SiO_2_ ink was manufactured using ink-jet 3D printing, which has 10 × 10 mm width and length. When manufactured in three-dimensional insulating layers with 30 layers of ink-jet printing, the x-axis was 10.34 mm, the y-axis was 10.42 mm, and the height was 174.3 μm, showing an error rate of 3.8% of the initial CAD (Computer Aided Design) design. This was judged to be a printing error that occurred because the spreads were faster than the speed at which the modified nano SiO_2_ ink was cured during the photopolymerization reaction. The photo-cured modified nano SiO_2_ insulation layer resistance, fabricated with the ink-jet 3D printing process, represented a value of 2.43 × 10^13^ Ω·cm.

### 3.4. Rheological Properties and Printability of IPL-Sinterable Nano Cu Ink

**Figure 6:**
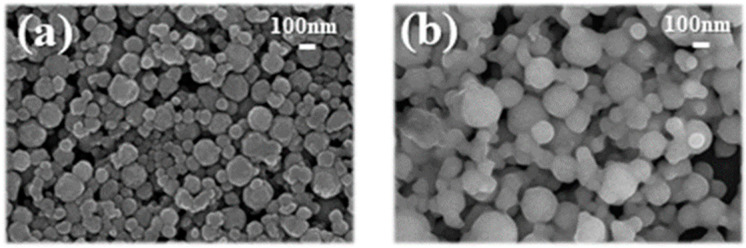
FE-SEM images of IPL-sinterable nano Cu ink: (**a**) w/o PVP and (**b**) with PVP.

A conductive layer with IPL-sinterable nano Cu ink was manufactured on the insulation layer of modified nano SiO_2_ ink using ink-jet 3D printing. In Figure 6, the FE-SEM results showed nano Cu particles with a particle size of about 100 nm. If PVP was not added in Figure 6a, Cu particles were agglomerated and were not uniformly sintered, causing peeling problems, which adversely affected the electrical and mechanical properties. As shown in Figure 6b, the problem could be solved by adding a capping agent such as PVP for the role of dispersion stabilizers and reduction aids [30,31,32]. Table 2 summarizes the rheological properties of the IPL-sinterable nano Cu inks used in this study. When PVP was added, jetting was impossible due to the viscosity value of 48.45. Thus, the surface tension, density, and viscosity were controlled through EtOH and DEG to obtain printability and spherical droplets. The reason why different solvents were used was that the edges evaporated first due to the difference in surface tension and concentration due to the coffee ring phenomenon. The internal liquid was used to prevent this phenomenon during the annealing process because it flowed to the edge and was concentrated at the highest concentration, causing problems in resolution [33].

**Figure 7:**
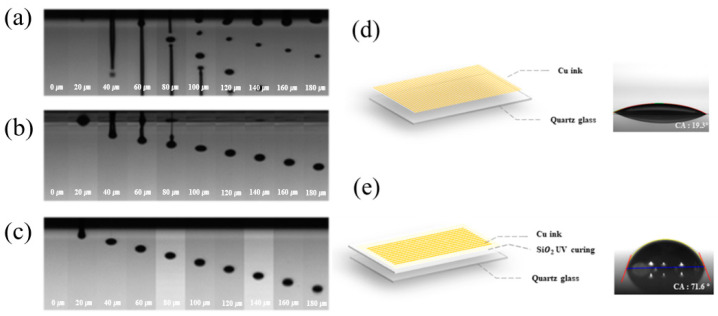
Jetting behavior of IPL-sinterable nano Cu ink; (**a**) before optimization; (**b**) after single-pulse optimization; (**c**) after multi-pulse optimization, contact angle, and ink-jet printed pattern of IPL-sinterable nano Cu ink; (**d**) on a quartz glass substrate; and (**e**) on photo-cured SiO_2_ insulation layer.

Figure 7 shows the jetting behavior of IPL-sinterable nano Cu ink before and after single-pulse/multi-pulse optimization. Figure 7a illustrates the jetting behavior of IPL-sinterable nano Cu ink before the optimization of the printing conditions, wherein the operating voltage was 78 V, the rising time and fall time was each 1 μs, the dwell time was 3 μs, and the delay time was 1 μs. In this operation, IPL-sinterable nano Cu ink was observed to be jetting unstable, the ink was a long tail, and the satellite drop problem occurred. In contrast, Figure 7b shows the well-defined droplet formation of IPL-sinterable nano Cu ink for single-pulse jetting conditions. The operation voltage was 78 V, the rising time and falling time were 5.2 μs, the dwell time was 13.2 μs, and the delay time was 1 μs. In the jetting of 0 to 80 μs, the IPL-sinterable nano Cu ink droplet was unstable, but after 100 μs, it could be seen that the spherical droplet was released stably. Figure 7c illustrates the jetting behavior of the IPL-sinterable nano Cu ink for multi-pulse operation conditions. The operation voltage of the first pulse was 80 V, the rising and falling times were 4 μs, and the dwell time was 12 μs, whereas the second-pulse operating voltage was 37 V, the rising and falling times were 1 μs, the dwell time was 3 μs, and the delay time was 1 μs. It was confirmed that the IPL-sinterable Cu nano ink had a stable spherical droplet form immediately after jetting under optimized multi-pulse conditions. The shorter the time it took for the ink from the printer head to be spherical, the narrower the distance was between the substrate and the head z-axis during printing, allowing the droplet during jetting to be reliably jetted into external variables [34,35,36]. Figure 7d,e show the contact angle of the ink-jet 3D printed conduction layer using IPL-sinterable nano Cu ink on top of the photo-cured nano SiO_2_ insulation layer and a quartz substrate. The contact angle of IPL-sinterable nano Cu ink on the quartz substrate and the photo-cured nano SiO_2_ insulation layer showed 19.3° and 71.6°, respectively.

**Figure 8:**
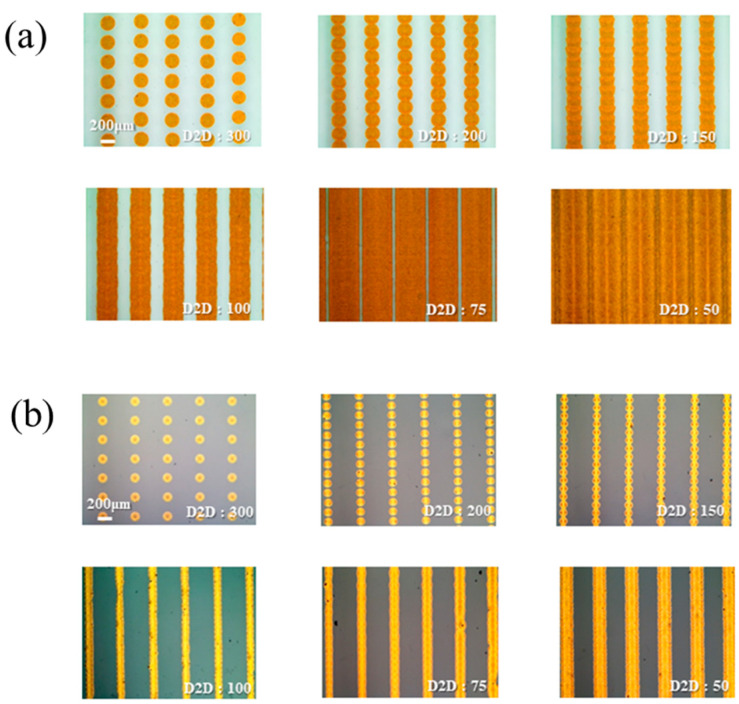
Ink-jet printed circuit according to D2D interval: (**a**) on a quartz glass substrate and (**b**) on photo-cured SiO_2_ insulation layer.

Figure 8 shows the microcircuit line width of the printed conductive nano Cu ink, controlling the D2D distance of 50 to 300 μm under optimized multi-pulse conditions. As shown in Figure 8a, the IPL-sinterable nano Cu ink printed on the quartz substrate had a single droplet width of 192.4 μm and was printed under the D2D 100 μm condition, but the outer contour of the line was confirmed to be uneven. In Figure 8b, showing the results from controlling the D2D distance of conductive nano Cu ink on top of the insulation layer, a single droplet size was 86.48 μm, and a line with a width of 109.01 μm was printed under the condition of D2D 100 μm. Since the ratio of solid material to an area of nano Cu ink in the printed droplet was the same, the thinner the line width of the printed conductive layer, the greater the number of particles in Cu, which could have excellent conductive characteristics.

### 3.5. Optimization of Annealing Temperature with IPL-Sinterable Nano Cu Ink

**Figure 9:**
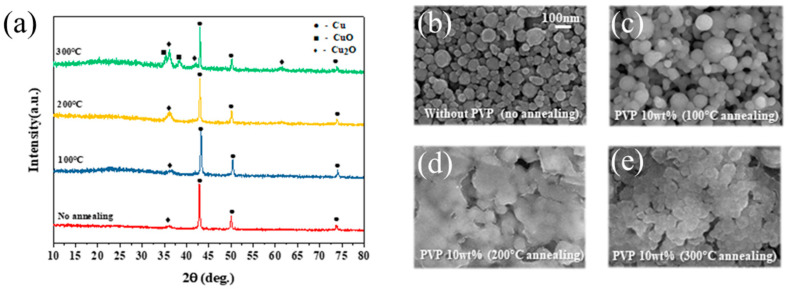
(**a**) XRD patterns of the ink-jet printed Cu conductive layer on a photo-cured SiO_2_ insulation layer after the annealing process and SEM images; (**b**) no annealing; (**c**–**e**) with PVP after annealing at 100 °C, 200 °C, and 300 °C.

Figure 9 shows the analysis results of XRD and SEM after annealing the Cu conductive layer at 100 °C, 200 °C, and 300 °C. In the IPL-sinterable nano Cu ink, since the particle size was small and the specific surface area was large, it was vulnerable to oxidation. Therefore, PVP was used as a surface stabilizer, dispersant, and capping agent. After printing, the annealing process could suppress cracking or peeling by removing the remaining solvent in the conductive layer of the Cu internal stress [37,38,39]. In the XRD analysis of Figure 9a, the peaks of pure Cu appeared at 43.8°, 50.6°, and 73.6°, the Cu_2_O peaks appeared at 36.4° and 61.7°, and the CuO peaks were 35.2° and 37.8°. As the annealing temperature increased, the metal Cu could be oxidized. Figure 9b–e show an SEM image of the surface of Cu nanoparticles without PVP, which is rough and agglomerated. The IPL-sinterable nano Cu ink with 10 wt% PVP after the annealing process. In Figure 9c, when annealed at 100 °C, re-oxidation by annealing treatment did not occur, and it was confirmed that the PVP was capping on the oxidation shell. In Figure 9d, after annealing the Cu particles at 200 °C, the Cu_2_O peak increased at 36.4°, which was oxidized because the PVP with a melting point of 150 °C was melted in the annealing process. From the results of Figure 9e, it could be seen that the PVP with a boiling point of 218 °C evaporated and then was re-oxidized by the annealing process. At the annealing process at 300 °C, the XRD diffraction peak intensity of Cu_2_O phase was increased, and 35.2° and 37.8° peaks of CuO and a new peak of Cu_2_O appeared at 61.7°. Since PVP acted as a reduction aid and anti-oxidant: if evaporated, it had a bad effect on the electrical properties of the Cu conductive layer. Therefore, it was confirmed that the best annealing temperature for the suitable IPL sintering process was 100 °C.

### 3.6. The Effect of Applied IPL Voltage to Sintering of Cu Conductive Layer

**Figure 10:**
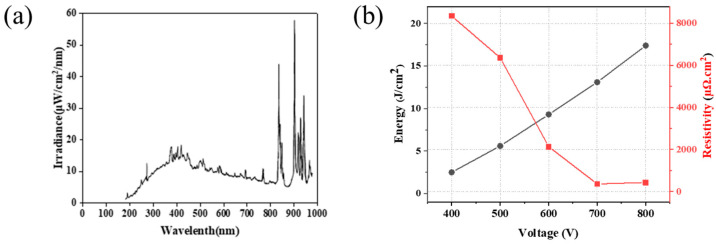
Typical instantaneous spectral distribution of (**a**) irradiance and (**b**) single-pulse energy generated by an IPL xenon lamp.

Figure 10 shows the irradiance and light energy irradiated per unit area of the xenon lamp used in the IPL sintering adopted in this study. As seen in Figure 10a, the irradiance was measured at wavelengths of 0 to 1000 nm and showed excellent ultraviolet areas from 800 to 1000 nm. Figure 10b measured energy using an energy meter when a voltage of 400 to 800 V was applied between a specimen and a Xenon lamp. The xenon lamp energy irradiated on substrates had energy ranging from 2.5 to 17.4 J/cm^2^, resulting in the effect of the applied voltage of the IPL sintering process on the electrical property of the Cu conductive layer. The resistance of the Cu conductive layer on the photo-cured SiO_2_ insulation layer was measured at 340 μΩ·cm (400 V), 120 μΩ·cm (500 V), and 63 μΩ·cm (600 V). However, under the applied voltage at 600 V, IPL sintered was not enough light energy of nanoparticle necked, indicating a relatively high resistance value. Further, the resistance to the electrical properties was 29 μΩ·cm and 55 μΩ·cm, as the applied voltage increased to 700–800 V of the Cu conductive layer. This slight increase of the Cu conductive layer’s electrical conductivity under the applied voltage of 800V was due to an excessive energy supply [40,41,42,43]. In conclusion, it was confirmed that the IPL sintering process could effectively remove the surface oxide of Cu nanoparticles and complete necking between Cu nanoparticles, resulting in superior electrical properties.

**Figure 11:**
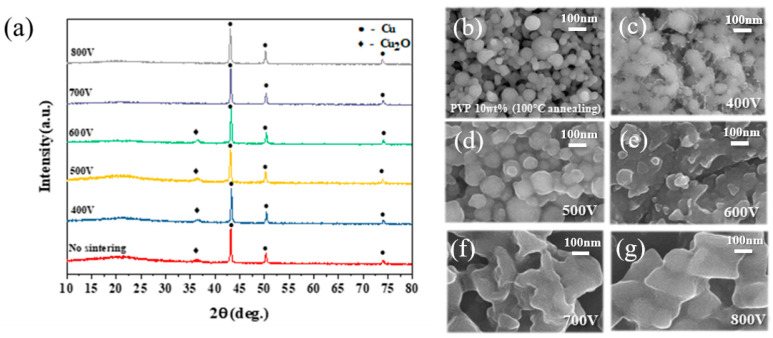
(**a**) XRD patterns of the ink-jet printed Cu conductive layer on photo-cured SiO_2_ insulation layer after the IPL sintering process and FE-SEM images; (**b**) no sintering; (**c**) 400 V; (**d**) 500 V; (**e**) 600 V; (**f**) 700 V; and (**g**) 800 V.

Figure 11 shows the analysis results of the XRD and SEM of the IPL-sintered Cu conductive layer after the IPL sintering process at the applied voltage of 400 to 800 V. As seen in Figure 11a, the XRD peak intensity of the Cu_2_O (36.4°) phase gradually decreased with increasing voltage to 600 V. Finally, the Cu_2_O (36.4°) was reduced entirely to under the IPL applied voltage at 700 V, confirming that the peak had disappeared. As shown in Figure 11b–g, as the applied voltage of IPL sintering increased from 400 V to 600 V, it was confirmed that the Cu nanoparticles were gradually necking [44,45]. Further, when IPL sintering was applied at voltages between 700 and 800 V, it could be seen that sufficient energy was supplied for the Cu nanoparticle sintering.

### 3.7. Electrical and Mechanical Properties of the Ink-Jet 3D Complex Structure

**Figure 12:**
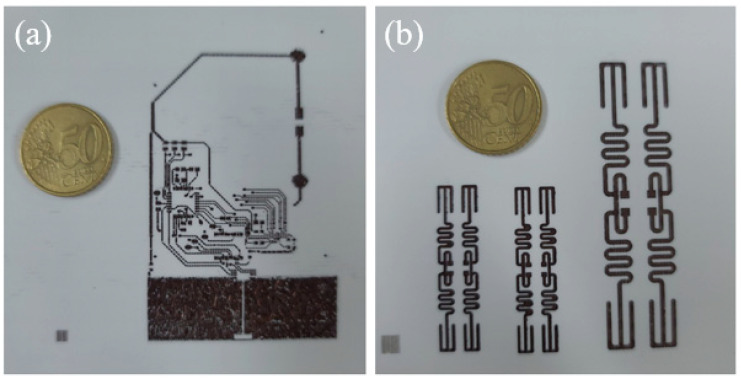
Photograph of ink-jet 3D printed complex structures with photo-cured SiO_2_ ink and IPL sintered Cu ink. The pattern of Cu ink is (**a**) PCB circuit (**b**) antenna circuit.

Table 3 shows the adhesion test results of the Cu conductive layer on the photo-cured nano SiO_2_ insulation layer after the IPL sintering process. The adhesion strength measurement was performed in three cycles for each sample, and, as shown in the results, the adhesion of the Cu conductive layer was confirmed to be affected by the increase in the applied voltage. As the applied voltage increased to 700 V, the adhesion strength of the Cu conductive layer on the photo-cured SiO_2_ insulation layer reached the best result (5B). In addition, circuits printed with optimized Cu ink patterning with excellent resolution without ink spreading. The final ink-jet 3D printed complex structure is shown in Figure 12a,b.

On the other hand, the adhesion strength tended to decrease significantly under 800 V conditions. This caused a duet of excessive energy irradiation, which enabled the complete necking of Cu particles but weakened the adhesion between the photo-cured SiO_2_ insulation layer and the Cu conductive layer, resulting in peeling. Therefore, it was confirmed that the Cu conductive layer had the best electrical conductivity and adhesion strength under the IPL voltage of 700 V.

## 4. Conclusions

In this study, a 3D complex structure with excellent electrical and mechanical properties was manufactured using multi-material ink-jet 3D printing. The ink-jet 3D printed structure was composed of an insulation part and a conductive part of which the insulation layer was made of photo-curable nano SiO_2_ ink, and the conductive layer was made of IPL-sinterable nano Cu ink. A 3D complex structure with excellent resolution could be produced by optimizing the rheological properties of both inks and their composition and printing conditions. A photo-curable nano SiO_2_ ink for the insulation layer was synthesized by mixing surface-modified nano SiO_2_ particles, and a photo-curable monomer and anionic surfactant were added to improve the contact angle and rheological property. The modified nano SiO_2_ ink had a contact angle characteristic of 70.9° on the PFTS hydrophobic coated substrate and showed a photopolymerization conversion rate of 65.3% in the UV wavelength of 385 nm. Finally, the ink-jet 3D printed insulation layer with the modified nano SiO_2_ had x- and y-axes of 10 mm and 174.3 μm z-axis and exhibited a resistance value of 2.43 × 10^13^ Ω·cm and an error rate of 3.8%. A conductive layer with IPL-sinterable nano Cu ink added PVP was manufactured on the insulation layer of modified nano SiO_2_ ink using ink-jet 3D printing, and DEG and ethanol were added to optimize the rheological properties and multi-pulse operating conditions to improve the printability of the ink. The uniform, continuous, and subtle Cu pattern was printed by adjusting the D2D to control the jetting distance of IPL-sinterable nano Cu ink. The printed Cu conductive layer was annealed at 100 °C to remove the solvent, and then IPL sintering was conducted at 700 V. The IPL-sintered Cu conductive layer on the photo-cured SiO_2_ insulation layer had a resistance value of 29 μΩ·cm and excellent adhesion properties of 5B. As a result, optimizing the rheological properties and operating conditions of the ink resulted in a superior resolution of the spherical droplet without satellite drop and spread ink. Finally, using the optimized photo-curable nano SiO_2_ ink and IPL-sinterable nano Cu ink added PVP, a complex 3D structure of the SiO_2_ insulation and Cu conductive layers with excellent resolution, mechanical, and electrical properties was successfully manufactured in the ink-jet 3D printing and IPL sintering.

## Figures and Tables

**Table 1:** Rheological properties of photo-curable nano SiO_2_ ink and modified nano SiO_2_ ink.

	Viscosity (mPas)	Density (g/mL)	Surface Tension (mN/m)	Inverse of Ohnesorge Number (z)
SiO_2_ ink	5.68	0.94	24.21	5.82
ModifiedSiO_2_ ink	4.83	0.98	27.91	7.50

**Table 2:** Rheological properties of IPL-sinterable nano Cu ink. These measurements were to ensure that the formulations had a Z value between 1 and 10 before printing.

Solvent Content (Weight Fraction)	Viscosity (mPas)	Density (g/mL)	Surface Tension (mN/m)	PVP (wt%)	Inverse of Ohnesorge Number (z)
Cu NP (0.40) + DEG (0.60)	23.12	1/39	34.39	0	1.83
Cu NP (0.40) + DEG (0.60)	48.45	1.45	34.78	10	1.07
Cu NP (0.20) + DEG (0.30) + EtOH(0.50)	4.54	1.22	29.46	0	9.73
Cu NP (0.20) + DEG (0.30) + EtOH (0.50)	9.21	1.23	30.22	10	4.84
Cu NP (0.20) + DEG (0.30) + EtOH (0.50)	24.13	1.25	31.96	20	1.85

**Table 3:** Adhesion strength results of the ink-jet printed conducting layer with IPL-sinterable nano Cu ink after IPL sintering (ASTM D3359).

	400 V			500 V			600 V			700 V			800 V		
Cycle	1	2	3	1	2	3	1	2	3	1	2	3	1	2	3
Adhesion	4B	3B	1B	4B	4B	3B	5B	5B	4B	5B	5B	5B	3B	1B	0B

## Data Availability

Data are available in the main text.
